# A Disposable Blood-on-a-Chip for Simultaneous Measurement of Multiple Biophysical Properties

**DOI:** 10.3390/mi9100475

**Published:** 2018-09-20

**Authors:** Yang Jun Kang

**Affiliations:** Department of Mechanical Engineering, Chosun University, 309 Pilmun-daero, Dong-gu, Gwangju 61452, Korea; yjkang2011@chosun.ac.kr; Tel.: +82-62-230-7052

**Keywords:** RBC deformability, RBC aggregation, hematocrit, multiple biophysical properties, microfluidic device, periodic on-off blood flow, blood velocity fields, image intensity of blood flow

## Abstract

Biophysical properties are widely used to detect pathophysiological processes of vascular diseases or clinical states. For early detection of cardiovascular diseases, it is necessary to simultaneously measure multiple biophysical properties in a microfluidic environment. However, a microfluidic-based technique for measuring multiple biophysical properties has not been demonstrated. In this study, a simple measurement method was suggested to quantify three biophysical properties of blood, including red blood cell (RBC) deformability, RBC aggregation, and hematocrit. To demonstrate the suggested method, a microfluidic device was constructed, being composed of a big-sized channel (BC), a parallel micropillar (MP), a main channel, a branch channel, inlet, and outlets. By operating a single syringe pump, blood was supplied into the inlet of the microfluidic device, at a periodic on-off profile (i.e., period = 240 s). The RBC deformability index (DI) was obtained by analyzing the averaged blood velocity in the branch channel. Additionally, the RBC aggregation index (AI_N_) and the hematocrit index (H_iBC_) were measured by analyzing the image intensity of blood flows in the MP and the BC, respectively. The corresponding contributions of three influencing factors, including the turn-on time (T_on_), the amplitude of blood flow rate (Q_0_), and the hematocrit (Hct) on the biophysical indices (DI, AI_N_, and H_iBC_) were evaluated quantitatively. As the three biophysical indices varied significantly with respect to the three factors, the following conditions (i.e., T_on_ = 210 s, Q_0_ = 1 mL/h, and Hct = 50%) were maintained for consistent measurement of biophysical properties. The proposed method was employed to detect variations of biophysical properties depending on the concentrations of autologous plasma, homogeneous hardened RBCs, and heterogeneous hardened RBCs. Based on the observations, the proposed method exhibited significant differences in biophysical properties depending on base solutions, homogeneous hardened RBCs (i.e., all RBCs fixed with the same concentration of glutaraldehyde solution), and heterogeneous hardened RBCs (i.e., partially mixed with normal RBCs and homogeneous hardened RBCs). Additionally, the suggested indices (i.e., DI, AI_N_, and H_iBC_) were effectively employed to quantify three biophysical properties, including RBC deformability, RBC aggregation, and hematocrit.

## 1. Introduction

Hemorheology is the study of the interactions between the blood and the vascular system [1]. Moreover, it deals with the rheological properties of vessel structures and blood components [2]. The rheological properties are determined by several factors such as hematocrit (Hct, the ratio of red blood cell (RBC) volume to total volume), the cell-free layer, the vessel diameter, and white blood cell (WBCs). As the number of RBCs is significantly higher than that of WBCs or platelets, the biophysical properties of the blood are dominantly determined by those of the RBCs. Specifically, biophysical studies are then focused on RBC-related properties, including Hct, blood viscosity, RBC deformability, and RBC aggregation. After the strong association between coronary heart diseases and biophysical properties has been reported [3], rheological properties have been widely used to detect the pathophysiological processes of vascular diseases or clinical states [4]. Gas transport and flow regulation in capillary vessels are significantly influenced by RBC deformability [4]. Owing to low cytoplasmic viscosity, high surface−volume ratio, and highly elastic membranes, RBCs have higher deformability [5]. Thus, individual RBCs can easily pass through capillary vessels whose diameters are much smaller than that of the RBCs [4]. RBC aggregation causes in vivo hemodynamic behaviors in low flow-rate regions of post-capillary venules to vary [4,6]. It is significantly elevated under inflammatory or pathophysiological conditions [7,8]. RBC aggregation strongly depends on several factors such as plasma proteins, membrane deformability, and Hct [9].

As a microfluidic device offers distinctive advantages, including fast response, a small volume of consumption, and promising point-of-care testing (POCT), the microfluidic-based technique has been widely adopted to quantify three biophysical properties such as RBC deformability, RBC aggregation, and Hct, as summarized in Table S1 (Supplementary Materials). First, RBC deformability has been quantified using several methods, including cell blockage [5,10,11,12], cell aspiration [13], and cell transit. In the cell transit technique, several parameters such as deformability index (DI) [14], cell margination [15], transit time [16,17], individual RBC velocity [18], and cell lysis [19] have been employed to measure the RBC deformability. Second, several methods including the photometric method [9,20], electric impedance [21], ultrasonic imaging [22], and microscopic imaging [23,24,25,26,27,28] have been suggested to quantify RBC aggregation. At last, Hct has been quantified by measuring the direct current response [29], electric resistance [30,31], the traveled distance of RBCs [32], and a histogram of grayscale intensity [33]. Previously, our group suggested simple methods for the simultaneous measurement of two biophysical properties (i.e., RBC deformability and viscosity [34], RBC deformability and viscoelasticity [35], and RBC aggregation and viscosity [27,36]). Specifically, blood viscosity has been obtained to monitor variations of Hct, because Hct has a strong influence on blood viscosity. Thus, two syringe pumps are required to supply blood and reference fluid at a specific flow rate [34,36]. When compared with blood viscosity measurement, Hct measurement becomes simple, because a single syringe pump is sufficient to supply blood. Simultaneous measurement of RBC aggregation and deformability are required to evaluate the contribution of RBCs or plasma proteins on biophysical properties. However, microfluidic-based techniques for measuring three biophysical properties, including RBC deformability, RBC aggregation, and Hct, have not been demonstrated. Thus, a new method should be devised to measure three biophysical properties, especially during blood flows, with a single syringe pump.

In this study, a simple measurement method has been adopted to quantify three biophysical properties of blood, such as RBC deformability, RBC aggregation, and Hct. To demonstrate the suggested method, a microfluidic device was composed of a big-sized channel (BC), a parallel micropillar (MP), a main channel, a branch channel, an inlet, and outlets. After the outlet of the MP was connected with a polyethylene tube, the end of the tube was completely clamped with a pinch valve. By operating a syringe pump, blood was supplied into the inlet of the microfluidic device in a periodic on–off profile. RBC deformability was then obtained by analyzing the averaged blood velocity in the branch channel. Additionally, RBC aggregation and Hct were measured by analyzing the image intensity of blood flows in the MP and BC, respectively.

The proposed method has several distinct advantages when compared to previous methods. First, the proposed method is able to measure three biophysical properties, including RBC deformability, RBC aggregation, and Hct. Specifically, the corresponding contributions of RBCs or plasma protein on the biophysical properties can be evaluated by measuring three properties, independently and simultaneously. Second, when measuring blood viscosity with co-flowing method [27], two syringe pumps are required to supply blood as test fluid and 1× PBS (phosphate-buffered saline) as a reference fluid. In this study, instead of blood viscosity, Hct is monitored under blood flows with a single syringe pump. Thus, the number of syringe pumps for fluid delivery is reduced from two to one.

As a demonstration, at first, the corresponding contributions of several factors (i.e., the on–off period, Hct, and blood flow rate) on the performance are quantitatively evaluated. Then, the method is employed to evaluate variations in biophysical properties with respect to the concentrations of plasma. Finally, the proposed method is used to detect homogeneous and heterogeneous RBCs. Here, heterogeneous RBCs are partially mixed by adding hardened RBCs into normal RBCs.

## 2. Materials and Methods

### 2.1. Blood Sample Preparation

In accordance with the ethics committee of Chosun University Hospital (CUH) (Chosun 2018-05-11), all experiments were performed by ensuring that the procedures were appropriate and humane. Concentrated RBCs were purchased from the Gwangju–Chonnam blood bank (Gwangju, Korea) and stored at 4 °C. When the storage time of RBCs exceeded seven days, all RBCs were disposed of properly. After the washing procedure of the concentrated RBCs was performed twice, blood samples were prepared by adding RBCs into specific base solutions. Subsequently, blood samples were kept at 4 °C before conducting experiments. All experiments were completed within 4 h.

In order to evaluate the effect of the Hct and base solution (i.e., diluted plasma) on the biophysical properties, the hematocrit (Hct) (Hct = 30%, 40%, and 50%) was prepared by adding normal RBCs into various concentrations of autologous plasma (C_plasma_) (C_plasma_ = 0, 30%, 50%, 70%, 85%, and 100%). Here, C_plasma_ = 0 and C_plasma_ = 100% denote PBS (phosphate-Buffered saline) solution (1×, pH 7.4, Gibco, Life Technologies, Seoul, Korea), and autologous plasma, respectively. The specific concentrations of plasma were prepared by diluting autologous plasma into the PBS solution. Next, in order to stimulate different degrees of RBC deformability, homogeneous hardened blood was prepared by adding RBCs fixed with the same concentrations of glutaraldehyde (GA) solution into autologous plasma. Five different concentrations of GA solution (C_GA_) (C_GA_ = 2 µL/mL, 4 µL/mL, 6 µL/mL, 8 µL/mL, and 10 µL/mL) were prepared by diluting pure GA solution (Grade II, 25% in H_2_O, Sigma-Aldrich, St. Louis, MO, USA) into the PBS solution properly. Normal RBCs were then hardened after dipping normal RBCs into the diluted GA solution for 10 min. Subsequently, homogeneous hardened blood (i.e., hardened RBCs suspended in PBS solution, Hct = 50%) was prepared by adding uniformly hardened RBCs to the PBS solution. Lastly, to detect the variations in biophysical properties with respect to the ratio of the hardened blood volume (V_h_) to the normal blood volume (V_n_), heterogeneous hardened blood with a mixing ratio (Ψ) (i.e., Ψ = V_h_/[V_h_ + V_n_], Ψ = 5%, 10%, 20%, 50%, and 100%) was prepared by adding the hardened blood into the normal blood partially. Specifically, normal blood (Hct = 50%) was prepared by adding normal RBCs into autologous plasma. Hardened blood (Hct = 50%) was prepared by adding RBCs fixed with a GA solution of C_GA_ = 2 µL/mL into autologous plasma.

### 2.2. Fabrication of a Microfluidic Device and Experimental Procedures

**Figure 1:**
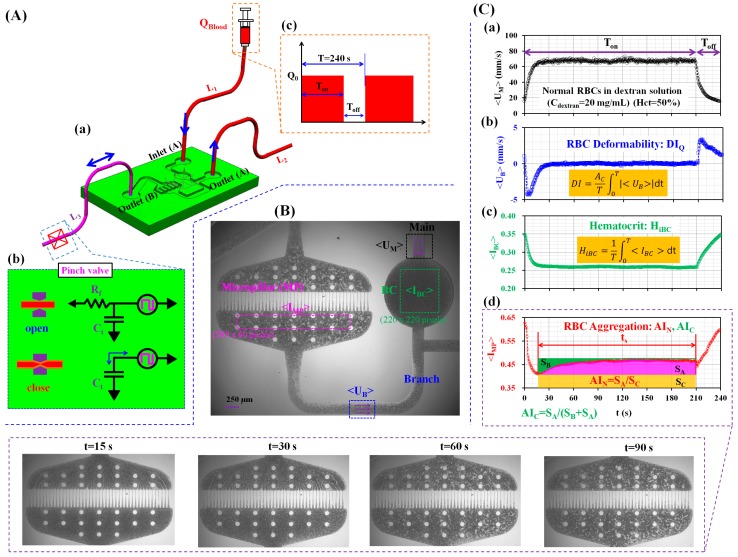
A proposed method for quantifying biophysical properties, including RBC (red blood cell) deformability, RBC aggregation, and hematocrit. (**A**) A schematic diagram of the proposed method, including a single syringe pump, a disposable microfluidic device, and a pinch valve. (**a**) The microfluidic device is composed of an inlet (**A**), two outlets (**A**,**B**), a big-sized channel (BC), a parallel micropillar (MP), a branch channel, and a main channel. Three polyethylene tubes (L_1_, L_2_, and L_3_) are connected to the inlet (**A**), and two outlets (**A**,**B**). (**b**) To induce back-and-forth blood flow in the MP, a pinch valve is employed to clamp a polyethylene tube connected to the outlet (**B**). Due to the tube compliance, blood flow is periodically oscillated depending on the blood flow-rate that is supplied from the syringe pump. (**c**) Blood flow-rate is set to a periodic on–off profile (i.e., amplitude of blood flow-rate = Q_0_, turn-on time = T_on_, turn-off time = T_off_, and period = T). (**B**) A high-speed camera triggered by a function generator is used at an interval of 0.5 s to consecutively capture two microscopic images at a frame rate of 5 kHz. Two averaged intensities (<I_MP_>, and <I_BC_>) and two averaged velocities (<U_B_>, and <U_M_>) are obtained by conducting digital image processing and a time-resolved micro-PIV technique, respectively. (**C**) As a preliminary demonstration, the hematocrit of blood was adjusted to 50% by adding normal RBCs into a dextran solution of C_dextran_ = 20 mg/mL. Blood was supplied into the inlet (**A**) at a periodic on–off profile (i.e., Q_0_ = 1 mL/h, T_on_ = 210 s, T_off_ = 30 s, and T = 240 s). (**a**) Temporal variations of the averaged blood velocity in main channel (<U_M_>) over a single period. (**b**) Temporal variations of the averaged blood velocity in the branch channel (<U_B_>) over a single period. DI is suggested to quantify RBC deformability. (**c**) Temporal variations of the averaged image intensity (<I_BC_>) in BC over a single period. H_iBC_ is proposed to quantify the hematocrit variations in the BC. (**d**) Temporal variations of the averaged image intensity (<I_MP_>) in the MP over a single period. AI_N_ and AI_C_ are adopted to evaluate the RBC aggregation. Sequential microscopic images depicted RBC aggregation in the MP with respect to time (t) (t = 15 s, 30 s, 60 s, and 90 s).

Figure 1(Aa) showed a schematic diagram of an experimental setup including a microfluidic device and a syringe pump. A microfluidic device for measuring three biophysical properties, such as RBC deformability, RBC aggregation, and Hct, was composed of a big-sized channel (BC, diameter = 1500 µm), a parallel micropillar (MP, N = 43, width = 4 µm, and length = 150 µm), a main channel (width = 250 µm), branch channel (width = 250 µm, and length = 4120 µm), an inlet, and outlets. The corresponding dimensions for the microfluidic channels are presented in the microscopic image, as shown in Figure S1 (Supplementary Materials). The channel depth of the microfluidic device was set to 4 μm.

A silicon master mold was fabricated using conventional micro-electromechanical-system fabrication techniques, such as photolithography and deep-reactive-ion etching. Polydimethylsiloxane (PDMS) (Sylgard 184, Dow Corning, Midland, MI, USA) was mixed with a curing agent at a ratio of 10:1. The PDMS mixture was poured onto the silicon master mold in a petri dish. Air bubbles dissolved in PDMS were completely removed by operating a vacuum pump for 1 hr. After curing the PDMS in a convective oven at 70 °C for 1 h, the PDMS block was peeled off from the silicon master mold. Subsequently, three ports (inlet and two outlets) were punched with a biopsy punch (outer diameter = 0.75 mm). After treating the PDMS block and the glass substrate with oxygen plasma (CUTE-MPR, Femto Science Co., Gyeonggi, Korea), a microfluidic device was fabricated by bonding the PDMS block to the glass substrate.

Two polyethylene tubes, L_1_ (length = 300 mm and inner diameter = 250 μm) and L_2_ (length = 200 mm and inner diameter = 250 μm), were tightly fitted to the inlet (A) and outlet (A). Additionally, L_3_ (length = 100 mms and inner diameter = 250 µm) was connected to the outlet (B). To remove air bubbles in the microfluidic channels and to avoid nonspecific binding of plasma proteins to the inner surface of the microfluidic channels, a BSA (bovine serum albumin) solution of C_BSA_ = 2 mg/mL was delivered through the outlet (A) using a disposable syringe.

After a time lapse of 5 min, all microfluidic channels were filled with PBS solution using a disposable syringe. Subsequently, the end of the tube connected to the outlet (B) was completely clamped with a pinch valve. As shown in Figure 1(Ab), when the tube is opened, blood flow passes through the tube. Because the tube provides a compliance effect, the fluidic circuit is modeled as fluidic resistance (R_f_) and tube compliance (C_t_), connected in parallel. However, when the end of the tube is completely clamped with the pinch valve, blood does not pass through the tube, and it oscillates back and forth. The fluidic circuit is then modeled as compliance (C_t_). Specifically, depending on the operation of the syringe pump (i.e., turn on or off), blood moves to the left and right directions, periodically. After a disposable syringe was filled with blood (~0.4 mL) and connected to the end of the tube fitted to the inlet (A), it was installed into the syringe pump. As shown in Figure 1(Ac), blood flow rate (Q_Blood_) was set to a periodic on-off profile (i.e., amplitude = Q_0_, turn-on time = T_on_, turn-off time = T_off_, and period (T) = 240 s).

The microfluidic device was positioned on an optical microscope (BX51, Olympus, Tokyo, Japan) equipped with a 4× objective lens (NA = 0.1). A high-speed camera (FASTCAM Mini, Photron, San Jose, CA, USA) was used to capture microscopic images of blood flows in the microfluidic channels. The camera had a spatial resolution of 1280 × 1024 pixels. Each pixel corresponded to 10 µm. With a function generator (WF1944B, NF Corporation, Tokyo, Japan), a pulse signal with period of 0.5 s triggered the high-speed camera. Two microscopic images were then sequentially captured at a frame rate of 5 kHz. All experiments were conducted at a room temperature of 25 °C.

### 2.3. Quantification of Image Intensity and Blood Velocity

As shown in Figure 1B, in order to measure the RBC deformability, RBC aggregation, and Hct, variations of four parameters (<U_B_>, <U_M_>, <I_MP_>, and <I_BC_>) were obtained by conducting a time-resolved micro-particle image velocimetry (PIV) technique and digital image processing with commercial software (MATLAB 2014a, MathWorks, Natick, MA, USA).

First, in order to evaluate the temporal variations of the averaged blood velocity (<U_M_> and <U_B_>), the corresponding region of interest (ROI) for the main and branch channels was selected as 60 × 120 and 120 × 60 pixels, respectively. Velocity fields were obtained by conducting a time-resolved micro-PIV technique. The size of the interrogation window was 16 × 16 pixels. The window overlap was 50%. The obtained velocity fields were validated with a median filter. The averaged velocities of blood flow in the main and branch channels (<U_M_> and <U_B_>) were calculated as an arithmetic average over the specific ROI.

Second, to evaluate the temporal variations in image intensity (<I_MP_> and <I_BC_>), the corresponding ROI for the MP and BC were selected as 585 × 60 and 220 × 220 pixels, respectively. The averaged image intensities in the MP and BC (<I_MP_> and <I_BC_>) were then obtained by calculating an arithmetic average of image intensity values over the specific ROI.

### 2.4. Simultaneous Measurement of RBC Deformability, RBC Aggregation, and Hct

In this study, three biophysical properties such as RBC deformability, RBC aggregation, and hematocrit were quantified by monitoring the blood velocity and image intensity in the microfluidic device.

In previous studies, the deformability of single RBC was quantified with several quantifiers such as the deformability index (DI), cell margination, transit time, individual RBC velocity, and cell lysis. By referring to the recent work conducted by Catarino et al. [37], the deformability index (DI) was found to be proportional to the RBC velocity for various degrees of RBCs, by measuring the RBC velocity and the deformed shape of RBCs. From this result, RBC velocity travelled in the microfluidic channel varied depending on the RBC deformability. When compared with their previous studies (i.e., extremely low hematocrit: 1~3%), the present method could measure the deformability of RBCs in a high-throughput fashion (i.e., hematocrit = 50%). As a working principle for the RBC deformability measurement [34,35,38], when supplying blood into a microfluidic device with parallel micropillars (MP) at constant blood flow rate, clogging of RBCs occurs in the MP over time. In other words, successive clogging of RBCs in the MP causes the averaged blood velocity in the branch channel to decrease significantly. Thus, blood velocity at outlet (A) varied depending on the blood velocity in the branch channel. Because the averaged blood velocity varied nonlinearly over time, the blood volume (ΔV) as the RBC deformability index was quantified by integrating the temporal variations of averaged blood velocity for a specific duration. The previous results indicated that the ΔV as the RBC deformability index was considered as being effective for monitoring the deformability of various RBCs, such as glutaraldehyde-based hardened RBCs [35,38], and malaria-infected RBCs [34], consistently.

When compared with the previous method, a microfluidic device proposed in this study was significantly improved to measure RBC deformability and RBC aggregation, especially in a simultaneous and periodic fashion. First, to minimize the effect of blood flow in the main channel on the blood flow in MP, a length of the branch channel was increased from 500 μm to 4120 μm. Second, to induce back-and-forth blood flow in the MP and the branch channel, a flexible tube filled with liquid was tightly fitted into the outlet (B) of the MP. After a certain amount of time, blood flow in the branch channel was completely stopped, even during constant blood delivery. Most bloods moved to outlet (A). Then, the RBCs in the MP were aggregated immediately at stasis. By turning off constant blood flow rate, the RBCs were disaggregated and moved to outlet (A), due to the compliance effect of the tube.

To induce turning-on blood delivery and turning-off blood delivery, a single syringe pump was employed to control the blood flow rate, at a periodic on–off profile. At last, because RBC aggregation varied depending on hematocrit, it was necessary to monitor the hematocrit during constant blood delivery. To effectively monitor variations of hematocrit over time, it was necessary to decrease the blood velocity significantly and to distribute the RBCs uniformly. As a simple idea, a big-sized circular channel (BC) (diameter = 1500 μm) was positioned in the main channel (width = 250 μm). According to the previous study [25], image intensity of blood varied depending on the channel width and channel depth. However, because all experiments were conducted with identical microfluidic devices, the device did not contribute to the varying hematocrit. In addition, the microscopic images were captured sequentially with the same image acquisition system. Image analysis was conducted consistently to calculate the image intensity of blood flows. Thus, the multiple factors should be controlled carefully for the consistent measurement of hematocrit. Then, variation of hematocrit was monitored by quantifying the image intensity of the RBCs selected within the BC.

As a preliminary demonstration, Hct was adjusted to 50% by adding the normal RBCs into a dextran solution of C_dextran_ = 20 mg/mL. Blood (i.e., normal RBCs suspended in dextran solution) was supplied into inlet (A) at a periodic on–off profile (i.e., Q_0_ = 1 mL/h, T_on_ = 210 s, T_off_ = 30 s, and T = 240 s).

First, as shown in Figure 1(Ca), temporal variations of <U_M_> were obtained over a single period. When the syringe was turned on, the <U_M_> remained at a constant value after 20 s. However, when the syringe was turned off, it decreased immediately.

Second, as illustrated in Figure 1(Cb), temporal variations of <U_B_> were obtained over a single period. During turn-on blood delivery, the blood moved to the left direction for up to 20 s. After 20 s, the blood flow was completely stopped. When turning off the syringe pump, the blood moved to the right direction immediately. According to previous studies [34,35], when the blood was supplied into the micropillar at a constant blood flow rate, the averaged blood velocity (<U>) was obtained by conducting a micro-PIV technique. After that, RBC deformability was quantified consistently by using blood volume (ΔV) (i.e., ΔV = $A_{c}\int_{0}^{t_{s}}Udt$). Here, A_c_ and t_s_ denoted the cross-sectional area of the microfluidic channel and the specific time, respectively. When compared with the previous method, the present method showed a negative sign of averaged blood velocity (<U_B_>) for turning-on blood delivery, and a positive sign of blood velocity for turning-off blood delivery. Referring to the previous method, positive and negative signs of <U_B_> indicated the degree of RBC deformability appropriately, depending on the turn-on or turn-off blood delivery. For this reason, absolute value of <U_B_> (i.e., |<U_B_>|) was employed to calculate RBC deformability index. In addition, because |<U_B_>| was obtained periodically, it was required to normalize |<U_B_>| for each period. Referring to the formula of the ΔV, DI as RBC deformability index was newly suggested as DI = $\frac{A_{C}}{T}\int_{0}^{T}\left|<U_{B}>\right|\mathrm{dt}$. Here, T represents the period.

Third, as shown in Figure 1(Cc), temporal variations of <I_BC_> were obtained over a single period. According to previous work [33], the intensity of grayscale image obtained at stationary blood flow varied significantly depending on Hct. Unlike the previous study, blood flow stopped or ran periodically. To reduce blood flow during turn-on blood delivery, the BC (diameter = 1500 µm) was positioned in the main channel (width = 250 µm). The variation of hematocrit in the microfluidic channel was monitored by analyzing the image intensity of blood flow in a specific ROI of the BC. As shown in Figure 1(Cc), <I_BC_> decreased during transient blood flow. Subsequently, it remained constant over turn-on time. After turning off the syringe pump, <I_BC_> increased gradually during turn-off blood delivery. RBC aggregation caused an increase in <I_BC_> under turning-off blood delivery. However, aggregated RBCs tended to be broken under the turning-on blood delivery. Thus, the <I_BC_> decreased gradually and remained constant. Two different behaviors of <I_BC_> were clearly visualized depending on the turning-on or turning-off blood delivery. For convenience, to quantify variations in Hct, the hematocrit index (H_iBC_) was calculated by averaging <I_BC_> over a single period (i.e., $H_{\mathrm{iBC}}=\frac{1}{T}\int_{0}^{T}<I_{\mathrm{BC}}>\mathrm{dt}$).

At last, as shown in Figure 1(Cd), temporal variations of <I_MP_> were obtained over a single period. The lower panels shows sequential microscopic images for representing RBC aggregation in the MP with respect to time (t) (t = 15 s, 30 s, 60 s, and 90 s). After 15 s, <I_MP_> increased continuously over time, due to RBC aggregation. By referring to previous studies [25,26], two RBC aggregation indices (AI_N_ and AI_C_) were employed to quantify the RBC aggregation. As shown in the inset of Figure 1(Cd), three factors (S_A_, S_B_, and S_C_) were calculated from variations of <I_MP_> obtained for a specific duration of t_s_ (i.e., syllectogram). Here, $S_{A}=\int_{t_{0}}^{t_{0}+t_{s}}\left(<I_{\mathrm{MP}}>-<I_{\mathrm{MP}}\left(t=t_{0}\right)>\right)\mathrm{dt},\;S_{B}=\int_{t_{0}}^{t_{0}+t_{s}}\left(<I_{\mathrm{MP}}\left(t=t_{0}+t_{s}\right)>-<I_{\mathrm{MP}}>\right)\mathrm{dt}$, and $S_{C}=\int_{t_{0}}^{t_{0}+t_{s}}<I_{\mathrm{MP}}\left(t=t_{0}\right)>)\mathrm{dt}$. Specifically, the conventional RBC aggregation index (AI_C_) was calculated as AI_C_ = S_A_/(S_B_ + S_A_) [39,40]. Furthermore, a new RBC aggregation index (AI_N_), which considered RBC sedimentation in the driving syringe [25,26], was calculated as AI_N_ = S_A_/S_C_.

## 3. Results and Discussion

### 3.1. Quantitative Evaluations of Three Factors (T_on_, Q_0_, and H_ct_) on the Biophysical Indices

The effect of three influence factors, such as turn-on time (T_on_), the amplitude of blood flow rate (Q_0_), and Hct on the biophysical indices (DI, AI_C_, AI_N_, and H_iBC_) was evaluated quantitatively by measuring four parameters (<U_M_>, <U_B_>, <I_BC_>, and <I_MP_>) over time.

**Figure 2:**
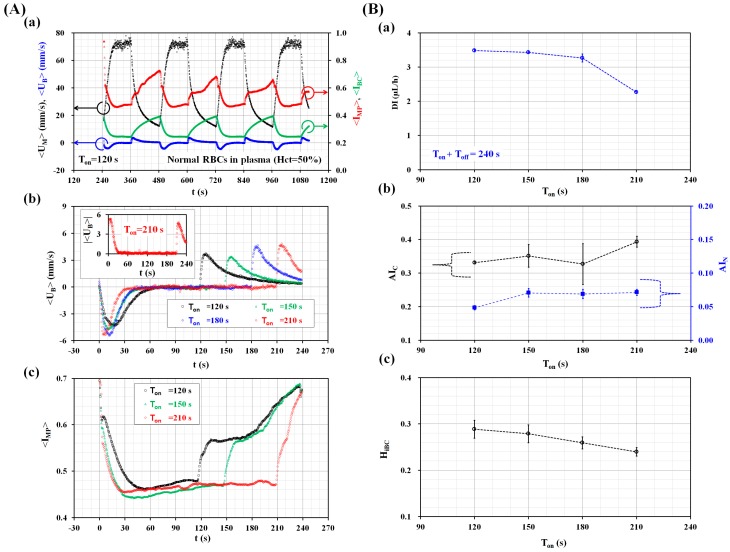
Quantitative evaluations of the effect of turn-on time (T_on_) on the four biophysical indices (i.e., DI, AI_C_, AI_N_, and H_iBC_). (**A**) Temporal variations of four parameters (<U_M_>, <U_B_>, <I_BC_>, and <I_MP_>). The hematocrit of blood was adjusted to 50% by adding normal RBCs into autologous plasma. (**a**) Temporal variations of averaged blood velocities (<U_M_>, and <U_B_>) and averaged image intensities (<I_BC_>, and <I_MP_>) at the turn-on time of T_on_ = 120 s. (**b**) Temporal variations of <U_B_> by increasing turn-on time (T_on_) (T_on_ = 120 s, 150 s, 180 s, and 210 s). Inset showed temporal variations of |<U>_b_| at the turn-on time of T_on_ = 210 s. (**c**) Temporal variations of <I_MP_> with respect to the turn-on time (T_on_) (T_on_ = 120 s, 150 s, and 210 s). (**B**) Variations of four biophysical indices (DI, AI_C_, AI_N_, and H_iBC_). (**a**) Variations of RBC deformability (DI) with respect to T_on_. (**b**) Variations of a conventional RBC aggregation index (AI_C_) and new RBC aggregation index (AI_N_) with respect to T_on_. (**c**) Variations of hematocrit index (H_iBC_) with respect to T_on_.

First, the effect of the turn-on time (T_on_) on three biophysical properties, including RBC deformability (DI), RBC aggregation (AI_C_ and AI_N_), and Hct (H_iBC_) was evaluated by measuring temporal variations of three parameters (<U_B_>, <I_BC_>, and <I_MP_>) over time. Here, Hct was adjusted to 50% by adding normal RBCs into autologous plasma. The period was fixed at T = 240 s. Figure 2(Aa) shows the temporal variations of the averaged blood velocities (<U_M_> and <U_B_>) and the averaged image intensities (<I_BC_> and <I_MP_>) at the turn-on time of 120 s. Depending on sequential turn-on and turn-off operation of the syringe pump, four parameters varied periodically over time. As shown in Figure 2(Ab), temporal variations of <U_B_> by increasing the turn-on time (T_on_) (T_on_ = 120 s, 150 s, 180 s, and 210 s) were obtained over a single period. Specifically, the inset shows temporal variations of |<U_B_>| at the turn-on time of 210 s. When T_on_ increased greatly, the maintained time of zero velocity (i.e., <U_B_> = 0) increased significantly. Figure 2(Ac) shows temporal variations of <I_MP_> with respect to T_on_. When T_on_ increased, RBC aggregation continued for a longer time. In other words, because the longer T_on_ increased the lasting time of the stationary blood flow in the MP, the longer turn-on time of 210 s caused <I_MP_> to increase significantly when compared with the shorter turn-on time of 120 s. As shown in Figure 2B, variations of the three biophysical indices were obtained by varying the turn-on time (T_on_). As shown in Figure 2(Ba), variations of DI were obtained by averaging |<U_B_>| with respect to T_on_. The DI decreased gradually for up to T_on_ = 180 s. Above T_on_ = 180 s, the DI decreased significantly. As shown in Figure 2(Bb), the RBC aggregation indices (AI_C_, and AI_N_) were obtained by analyzing <I_MP_> with respect to T_on_. A conventional RBC aggregation index (AI_C_) exhibited larger scattering and remained constant with respect to T_on_. However, above T_on_ = 120 s, the new RBC aggregation index (AI_N_) remained constant, irrespective of T_on_. As shown in Figure 2(Bc), H_iBC_ was obtained by analyzing <I_BC_> with respect to T_on_. H_iBC_ decreased gradually with an increase in turn-on time. From the results, it was found that four biophysical indices had been influenced by the turn-on time. Thus, in the following experiments, the turn-on-time of periodic blood flow was fixed to 210 s (i.e., T_on_ = 210 s, T_off_ = 30 s, and T = 240 s), especially for a consistent measurement of biophysical properties.

**Figure 3:**
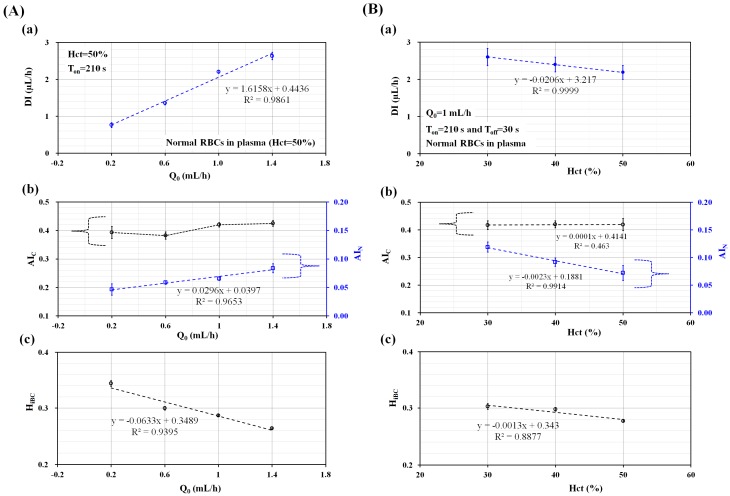
Quantitative evaluation of the effect of hematocrit (Hct) and amplitude of blood flow-rate (Q_0_) on biophysical properties. The hematocrit of blood was prepared by adding normal RBCs into autologous plasma. A syringe pump supplied the blood into a microfluidic device at a periodic on–off profile (i.e., Q_0_ = 1 mL/h, T_on_ = 210 s, and T = 240 s). (**A**) The effect of the amplitude of blood flow-rate (Q_0_) on variations of biophysical properties. (**a**) Variations of DI with respect to Q_0_ = 0.2 mL/h, 0.6 mL/h, 1 mL/h, and 1.4 mL/h. (**b**) Variations of AI_C_ and AI_N_ with respect to Q_0_. (**c**) Variations of H_iBC_ with respect to Q_0_. (**B**) The effect of hematocrit on variations of the biophysical properties. (**a**) Variations of DI with respect to Hct = 30%, 40%, and 50%. (**b**) Variations of RBC aggregation indices (AI_C_, and AI_N_) with respect to Hct. (**c**) Variations of H_iBC_ with respect to Hct.

Second, the effect of amplitude of periodic blood flow rate (Q_0_) on variations of biophysical indices was quantitatively evaluated. Here, the turn-on time was set to 210 s (T_on_ = 210 s). Hct was adjusted to 50% by adding normal RBCs into autologous plasma. As shown in Figure 3(Aa), variations of DI were obtained with respect to Q_0_ = 0.2 mL/h, 0.6 mL/h, 1 mL/h, and 1.4 mL/h. DI increased linearly by increasing Q_0_. According to a linear regression analysis conducted with Excel^TM^ (Microsoft, Redmond, WA, USA), the coefficient of linear regression yielded a higher value of R^2^ = 0.9861. Thus, the DI was strongly influenced by the amplitude of periodic blood flow rate (Q_0_). As shown in Figure 3(Ab), variations of AI_C_ and AI_N_ were obtained by increasing Q_0_ from Q_0_ = 0.2 mL/h to Q_0_ = 1.4 mL/hr. Here, the AI_C_ was determined dominantly by the variation of <I_MP_>. However, the AI_N_ varied depending on the variation and minimum value of <I_MP_> (i.e., <I_MP_>_min_) for specific period of t_s_. For this reason, the AI_C_ remained constant irrespective of Q_0_. According to linear regression analysis between AI_N_ and Q_0_, a higher value of R^2^ = 0.9395 was obtained. In other words, the AI_N_ increased linearly with respect to Q_0_, because S_C_ (i.e., S_C_ = <I_MP_>_min_ × t_s_) [25] decreased significantly. From the results, it was found that the three biophysical indices (DI, AI_N_, and H_iBC_) varied linearly with respect to Q_0_. In the following experiments, blood flow rate (Q_0_) was fixed to Q_0_ = 1 mL/h, especially for consistent measurement of biophysical properties.

At last, the effect of Hct on variations of biophysical indices was quantitatively evaluated. Here, Hct was adjusted to 30%, 40%, and 50% by adding normal RBCs into autologous plasma. A syringe pump was set to a periodic on–off profile (i.e., Q_0_ = 1 mL/h, T_on_ = 210 s, and T_off_ = 30 s) for supplying blood into a microfluidic device. As shown in Figure 3(Ba), variations of DI were obtained with respect to Hct. According to the linear regression analysis, the DI decreased linearly with respect to Hct, because the coefficient of the linear regression yielded a higher value of R^2^ = 0.9999. From the results, for the consistent measurement of RBC deformability, Hct should be fixed at a constant value. As shown in Figure 3(Bb), variations of AI_C_ and AI_N_ were obtained with respect to Hct. AI_C_ remained constant with respect to Hct (i.e., R^2^ = 0.463). However, AI_N_ decreased linearly with respect to Hct (i.e., R^2^ = 0.9914). From this result, the AI_N_ was considered to be an effective index for evaluating the effect of Hct when compared with AI_C_. As shown in Figure 3(Bc), variations of H_iBC_ were obtained with respect to Hct. As the linear regression analysis yielded a higher value of R^2^ = 0.8877, H_iBC_ showed a strong relationship to Hct. Thus, H_iBC_ can be employed to monitor Hct.

### 3.2. Performance Evaluation of the Proposed Method

The proposed method was employed to detect variations in biophysical properties depending on the concentrations of autologous plasma, homogeneous hardened RBCs, and heterogeneous hardened RBCs. Here, blood was supplied into a microfluidic device at a periodic on–off profile (i.e., Q_0_ = 1 mL/h, T_on_ = 210 s, and T_off_ = 30 s).

**Figure 4:**
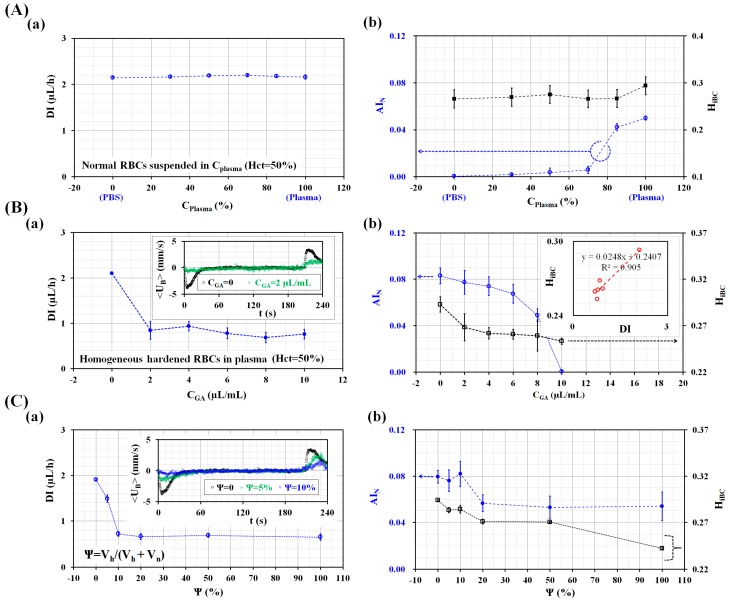
Performance evaluation of the proposed method. (**A**) Variations of blood biophysical properties with respect to the concentration of plasma (C_plasma_). Blood was supplied into a microfluidic device at a periodic on–off profile (i.e., Q_0_ = 1 mL/h, T_on_ = 210 s, and T_off_ = 30 s). The hematocrit of blood was adjusted by normal RBCs into a specific concentration of plasma. (**a**) Variation of DI with respect to C_plasma_ = 0, 25%, 50%, 70%, 85%, and 100%. (**b**) Variations of AI_N_ and H_iBC_ with respect to C_plasma_. (**B**) Detection of homogeneous hardened blood composed of homogeneous hardened RBCs with GA solution. The hematocrit of hardened blood was adjusted to 50% by adding hardened RBCs into autologous plasma. (**a**) Variations of DI with respect to C_GA_ = 0, 2 µL/mL, 4 µL/mL, 6 µL/mL, 8 µL/mL, and 10 µL/mL. (**b**) Variations of AI_N_ and H_iBC_ with respect to C_GA_. (**C**) Detection of heterogeneous blood composed of normal RBCs and partially hardened RBCs. Here, normal RBC was hardened by dipping them into 2 µL/mL GA solution. Then, the hematocrit of hardened blood and normal blood were adjusted to 50% by adding hardened RBCs and normal RBCs into autologous plasma. Specifically, a mixing ratio (Ψ) was defined as the ratio of hardened blood volume to total blood volume (i.e., Ψ = V_h_/[V_h_ + V_n_]). Here, V_h_ and V_n_ denote hardened blood and normal blood, respectively. (**a**) Variations of DI with respect to Ψ = 0, 5%, 10%, 20%, 50%, and 100%. (**b**) Variations of AI_N_ and H_iBC_ with respect to Ψ.

First, variations of biophysical properties were obtained with respect to the specific concentration of autologous plasma (C_plasma_) (C_plasma_ = 0, 25%, 50%, 70%, 85%, and 100%). Here, C_plasma_ = 0 and C_plasma_ = 100% denote pure PBS solution and pure autologous plasma, respectively. Hct was adjusted to 50% by adding normal RBCs into the specific concentration of plasma. As shown in Figure 4(Aa), variations of DI were obtained with respect to C_plasma_. The DI remained constant irrespective of the autologous plasma concentration. In other words, the deformability of normal RBCs remained the same irrespective of the PBS solution or autologous plasma. The result exhibited similar trends with respect to the concentration of plasma when compared with that which was obtained by the previous study [34]. As shown in Figure 4(Ab), variations of AI_N_ and H_iBC_ were obtained with respect to the concentration of autologous plasma. The AI_N_ increased gradually for up to C_plasma_ = 70%. Above that, it increased significantly by increasing the concentration of autologous plasma. In other words, RBC aggregation enhanced significantly at higher concentration of autologous plasma. However, H_iBC_ remained constant irrespective of C_plasma_. This result indicated that Hct can be monitored consistently without considering the concentration of autologous plasma.

Second, the proposed method was employed to detect variations of biophysical properties for hardened blood composed of homogeneous hardened RBCs. Normal RBC was hardened by dipping into a specific concentration of GA solution (C_GA_) for 10 min. Thereafter, Hct was adjusted to 50% by adding hardened RBCs into plasma. As shown in Figure 4(Ba), variations of DI were obtained with respect to C_GA_ = 0, 2 µL/mL, 4 µL/mL, 6 µL/mL, 8 µL/mL, and 10 µL/mL. Here, C_GA_ = 0 µL/mL denotes PBS solution. Inset showed temporal variations of <U_B_> for normal RBCs and hardened with 2 µL/mL, over a single period of 240 s. When compared with the normal RBCs, the <U_B_> of hardened RBCs decreased significantly. Hardened RBCs caused the DI to decrease significantly. Additionally, the DI remained constant irrespective of C_GA_ = 2 µL/mL, 4 µL/mL, 6 µL/mL, 8 µL/mL, and 10 µL/mL. In other words, the GA solution of C_GA_ = 2 µL/mL reduced the RBC deformability significantly. The hardened RBCs were sufficiently detected by using the proposed method. Simultaneously, as shown in Figure 4(Bb), variations of AI_N_ and H_iBC_ were obtained with respect to C_GA_. The AI_N_ decreased gradually by increasing C_GA_. Taking into account the fact that the GA solution contributed to the decreasing RBC deformability, RBC aggregation decreased significantly due to the decrease in RBC deformability. Normal RBCs gave a higher value of AI_N_ = 0.083 ± 0.007. However, the hardened RBCs with C_GA_ = 10 µL/mL did not include RBC aggregation because AI_N_ gave a lower value of AI_N_ = 0.001 ± 0.001. The H_iBC_ showed a significant difference between normal RBCs (C_GA_ = 0) and hardened RBCs (C_GA_ = 2 µL/mL). Above C_GA_ = 2 µL/mL, it remained constant irrespective of C_GA_. As shown in the inset of Figure 4(Bb), this trend of H_iBC_ was very similar to that of DI because linear regression analysis yielded a higher value of R^2^ = 0.905. The results indicated that the Hct index (H_iBC_) was varied by the degree of RBC deformability.

At last, the proposed method was employed to detect heterogeneous RBCs composed of normal RBCs and partially hardened RBCs. Here, normal RBCs were hardened by dipping them into a GA solution of C_GA_ = 2 µL/mL. Then, the Hct of the hardened blood was adjusted to 50% by adding the hardened RBCs into autologous plasma. In addition, Hct of normal blood was adjusted to 50% by adding normal RBCs into autologous plasma. A mixing ratio (Ψ) was defined as the ratio of the hardened blood volume to the total blood volume (i.e., Ψ = V_h_/[V_h_ + V_n_]). Here, V_h_ and V_n_ denote the hardened blood volume and the normal blood volume, respectively. As shown in Figure 4(Ca), variations of DI were obtained with respect to the mixing ratio (Ψ) (Ψ = 0, 5%, 10%, 20%, 50%, and 100%). The Inset shows the temporal variations of <U_B_> with respect to Ψ = 0, 5%, and 10%. <U_B_> decreased significantly by increasing the mixing ratio. As shown in Figure 4(Ca), DI decreased significantly for up to Ψ = 10%. After that (i.e., Ψ > 10%), DI remained constant irrespective of Ψ. From the results, the proposed method detected heterogeneous normal RBCs included with 10% hardened RBCs. As shown in Figure 4(Cb), variations of AI_N_ and H_iBC_ were obtained with respect to the mixing ratio. The AI remained constant for up to Ψ = 10%. After that (i.e., Ψ > 10%), it decreased significantly and remained constant with respect to Ψ. From the result, RBC aggregation decreased significantly when normal RBCs were mixed with above 10% hardened RBCs. However, H_iBC_ decreased gradually by increasing the volume fraction of the hardened RBCs. As the hardened RBCs decreased H_iBC_ significantly, the Hct of hardened blood could be overestimated.

Based on the results of the experimental demonstrations, the proposed method exhibited significant differences in biophysical properties depending on the base solutions, the homogeneous hardened RBCs, and the heterogeneous hardened RBCs. Additionally, the suggested indices (i.e., DI, AI_N_, and H_iBC_) were effectively employed to quantify three biophysical properties, including RBC deformability, RBC aggregation, and Hct. From the experimental data obtained in the study, DI as the deformability index varied from DI = 0.759 (strongly hardened RBCs) to DI = 2.1 (Normal RBCs). In addition, H_iBC_ as hematocrit index varied from H_iBC_ = 0.277 (Hct = 30%) to H_iBC_ = 0.302 (Hct = 50%). At last, AI_N_ varied from AI_N_ = 0 (Hardened RBCs in PBS) to AI_N_ = 0.119 (Normal RBCs in plasma). Since the measurement quantifiers such as <U_B_>, <I_BC_>, and <I_MP_> vary depending on the microfluidic device and the blood flow rate, different values of several suggested indices are obtained from the specific expressions of multiple biophysical properties give Thus, it is necessary to check the variation ranges of several indices.

## 4. Conclusions

In this study, a simple measurement method was suggested to quantify three biophysical properties of blood, such as RBC deformability, RBC aggregation, and hematocrit. To demonstrate the suggested method, a microfluidic device was composed of a big-sized channel (BC), a parallel micropillar (MP), a main channel, a branch channel, an inlet, and outlets. By operating a syringe pump, blood was supplied into the inlet of the microfluidic device, at a periodic on-off profile (i.e., period = 240 s). RBC deformability index (DI) was obtained by analyzing the averaged blood velocity in the branch channel. Additionally, RBC aggregation indices (AI_N_, and AI_C_) and hematocrit index (H_iBC_) were measured by analyzing image intensity of blood flows in the MP and BC, respectively. The corresponding contributions of three influence factors, such as turn-on time (T_on_), amplitude of blood flow rate (Q_0_), and Hct on the biophysical indices (DI, AI_N_, and H_iBC_) were evaluated quantitatively. As the three biophysical indices varied significantly with respect to the three factors, the following condition (i.e., T_on_ = 210 s, Q_0_ = 1 mL/h, and Hct = 50%) was consistently employed for measurement of biophysical properties. As the demonstration, the proposed method was employed to detect variations of biophysical properties depending on the concentrations of autologous plasma, homogeneous hardened RBCs, and heterogeneous hardened RBCs. First, the DI and H_iBC_ remained constant irrespective of the concentration of autologous plasma. However, AI_N_ was enhanced significantly at a higher concentration of autologous plasma. Second, the proposed method detected a difference in RBC deformability for normal RBC and homogeneous hardened RBCs with GA solution of 2 µL/mL. The AI_N_ and H_iBC_ varied by the degree of RBC deformability. Lastly, DI and AI_N_ exhibited significant differences for heterogeneous normal blood with 10% hardened RBCs. H_iBC_ decreased gradually by increasing the volume fraction of hardened RBCs. From the experimental demonstrations, the suggested indices (i.e., DI, AI_N_, and H_iBC_) were effectively employed to quantify three biophysical properties, including RBC deformability, RBC aggregation, and Hct. Future tests will involve employing the proposed method to evaluate multiple biophysical properties under in vitro closed circulation, in a simultaneous and continuous fashion.

## Supplementary Materials

The following are available online at http://www.mdpi.com/2072-666X/9/10/475/s1, Table S1: Summary of microfluidic-based methods suggested for measuring three biophysical properties such as RBC deformability, RBC aggregation, and hematocrit. Figure S1: Specific dimensions of the microfluidic device used for the simultaneous measurement of multiple biophysical properties.
