# Supplementary material for: A Disposable Blood-on-a-Chip for Simultaneous Measurement of Multiple Biophysical Properties
Source: Micromachines (Basel). 2018 Sep 20;9(10):475. doi: 10.3390/mi9100475 (PMC6215101; doi:10.3390/mi9100475)
Supplement: Supplementary file 1 [file micromachines-09-00475-s001.pdf]

micromachines

Supplementary Materials

A Disposable Blood-on-a-Chip for Simultaneous Measurement of Multiple Biophysical Properties

Yang Jun Kang

Department of Mechanical Engineering, Chosun University, 309 Pilmun-daero, Dong-gu, Gwangju 61452, Korea; yjkang2011@chosun.ac.kr; Tel.: +82-62-230-7052

Table 1. Summary of microfluidic-based methods suggested for measuring three biophysical properties such as RBC deformability, RBC aggregation, and hematocrit.

**Blood Biophysical Properties**			**Multiple Measurements**	**Comments**	**Ref.**
**RBC Deformability**	**RBC Aggregation**	**Hematocrit**			
Cell blockage	X	X	X	-Malaria-infected RBCs<br>-Minimum diameter in taper channel[10]<br>-Clogging in single channel[5] or multiple channels[11,12]	[5,10–12]
Cell aspiration	X	X	X	-Malaria-infected RBCs<br>-Funnel single channel	[13]
Cell transit	X	X	X	-WBCs or malaria-infected RBCs<br>-Deformability index[14], cell margination[15], transit time[16,17], RBC velocity[18], and cell lysis[19]	[14–19]
	X	X	Deformability and viscosity[12] (viscoelasticity)[13]	-Multiple pillar channels<br>-Blood velocity or image intensity<br>-Two syringe pumps[34, 35]<br>-High throughput (Hct=50%)	[34–37]
X	Photometric method	X	X	-Scattering or transmission<br>-Pinch valve[9] or vacuum pump[20]	[9,20]
X	Electric impedance	X	X	-Conductivity<br>-Pipette (single drop blood)	[21]
X	Ultrasonic images	X	X	-Speckle size and<br>-Syringe pump (on-off)	[38]
X	Microscopic images	X	X	-Aggregation size[23]<br>-Cluster size or occurrence[24]	[23,24]
X		X	X	-Image intensity (RBC aggregation)<br>-Air-suction pump[25] or syringe pump (on-off)[26]	[25,26]
X		X	Aggregation and viscosity	-Image intensity<br>-Two syringe pumps (on-off)	[27,36]
X	X	DC current	X	-Current response under 100 V_{DC}	[29]
X	X	AC impedance	X	-Resistance ratio[30]<br>-Electric resistance in blood[31]	[30,31]
X	X	Traveled distance	X	-Paper-based microfluidic device (POCT)	[32]
X	X	Histogram of grayscale intensity	X	-Smartphone-based detection (POCT)	[33]

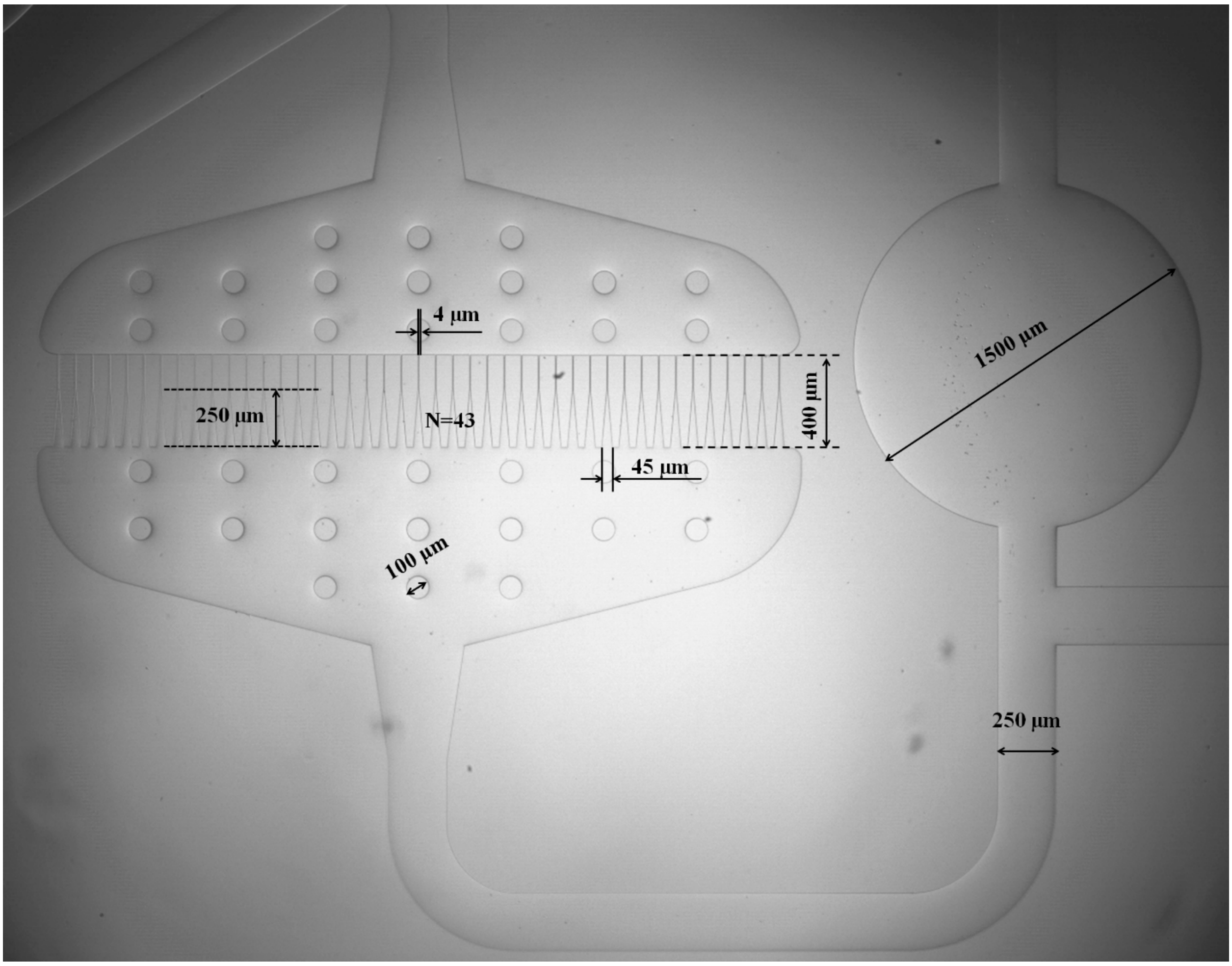

Figure S1. Specific dimensions of the microfluidic device for simultaneous measurement of multiple biophysical properties. .
